# Distribution of HPV types associated with cervical cancers in Scotland and implications for the impact of HPV vaccines

**DOI:** 10.1038/sj.bjc.6605556

**Published:** 2010-02-09

**Authors:** K Cuschieri, D H Brewster, A R W Williams, D Millan, G Murray, S Nicoll, J Imrie, A Hardie, C Graham, H A Cubie

**Affiliations:** 1 Scottish HPV Reference Laboratory, Specialist Virology Centre, Royal Infirmary of Edinburgh, Edinburgh EH16 4SA, UK; 2 Scottish Cancer Registry, Information Services Division, NHS National Services Scotland, Edinburgh EH12 9EB, UK; 3 Department of Pathology, Royal Infirmary of Edinburgh, Edinburgh EH16 4SA, UK; 4 Department of Pathology, Glasgow Royal Infirmary, Glasgow G4 OSF, UK; 5 Department of Pathology, Aberdeen Royal Infirmary, Aberdeen AB25 2ZN, UK; 6 Department of Pathology, Ninewells Hospital, Dundee DD1 9SY, UK; 7 Department of cytopathology, Monklands General Hospital, Airdrie ML6 OJS, UK; 8 Epidemiology and Statistics Core, Wellcome Trust Clinical Research Facility, Edinburgh EH4 2XU, UK

**Keywords:** HPV, genotyping, Scotland

## Abstract

**Background/metho:**

This study evaluated human papillomavirus (HPV) type prevalence in 370 Scottish invasive cervical cancers (ICCs) using HPV genotyping and HPV mRNA detection.

**Results:**

HPV 16 and/or 18 was detected in 72% of cancers overall and in 82% of HPV-positive cancers. HPV 45 and 16 were the most frequently transcribed types.

**Conclusion:**

A significant reduction in ICC in Scotland should be achieved through the HPV immunisation programme.

Human papillomavirus immunisation is being introduced in many countries across the world. In Scotland, a national school-based immunisation programme began in September 2008 for girls aged 12–17 years using HPV bivalent vaccine. The vaccine protects against infection with high-risk HPV 16 and 18 that are thought to contribute to around 70% of cervical cancers (http://www.fightcervicalcancer.org.uk, 2009). However, the data used to inform this percentage are based on worldwide meta-analyses, which have incorporated little UK, let alone Scottish, data (Clifford *et al*, 2003; Smith *et al*, 2007).

We undertook a sufficiently powered, systematic interrogation of cervical cancer cases from all regions of Scotland to determine HPV type-specific prevalence. This study was necessary to gauge the potential impact of the immunisation programme, which so far has achieved high uptake (93.3% for first dose and 89.5% for second dose) for girls in second year of secondary school (http://www.isdscotland.org/isd/5922.html, 2009).

The study was also undertaken to define a baseline prevalence against which to monitor changes over time in HPV types associated with cervical cancers. Acquisition of related demographic, clinical and HPV type-specific data allowed us to assess the relationship between HPV 16/18 status, disease stage (FIGO), disease grade and the expression profile of HPV types within cancers with multiple infections.

## Materials and methods

### Sample collection

A representative population-based sample of Scottish invasive cervical cancers (ICC) was achieved by selecting cases regionally from Edinburgh, Dundee, Aberdeen, Glasgow and Lanarkshire. Each site was asked to generate a sequential list of ICC cases, diagnosed from 2004 working backwards. 2004 was chosen as the most recent year for which data in the Scottish Cancer Registry were complete. The sample included both microinvasive and frank cancers, but not recurrent cancers. Review of cases was performed at each site and the relevant formalin-fixed paraffin-embedded block from the primary invasive tumour was sent to the Scottish HPV Reference Laboratory for sectioning and HPV testing.

### Data collection

Demographic and clinical information was sent to the Scottish Cancer Registry where laboratory and registry data were linked, with a view to attaching demographic variables (including the Scottish Index of Multiple Deprivation), prognostic variables (FIGO stage and ICD-O grade of differentiation) and a unique linkage number (to allow dates of death, when applicable, to be added at a later date). Where necessary, data cleansing was performed.

### HPV DNA genotyping

Typing of HPV was performed using HPV INNO-LiPA (Innogenetics NV, Gent, Belgium) according to the manufacturer's instructions. This is a very sensitive DNA-based assay that amplifies a 65-nucleotide fragment of the HPV L1 gene. Briefly, 10 *μ*m paraffin sections cut with a clean microtome knife were digested overnight with proteinase K. Crude lysate (10 *μ*l) was subjected to PCR and amplicons were hybridised to genotyping arrays capable of detecting high-risk HPV types and several low-risk types. A result of HPV ‘X’ was generated if the sample was HPV positive but could not be attributed to a type within the detection range of the assay.

### HPV mRNA detection within multiple infection

Any cancer that tested positive for >1 type, tested positive for low-risk type(s) only or generated a result of HPV X alone was subjected to mRNA E6/E7 expression analysis, using HPV PreTect RNA Proofer (NorChip AS, Klokkarstna, Norway) according to the manufacturer's instructions. The method involves RNA extraction before isothermal NASBA RNA amplification of full-length E6/E7. Type-specific detection was performed using molecular beacons corresponding to HPV types 16, 18, 31, 33 and 45.

### Data analysis

Our hypothesis was that HPV 16 and 18 distributions in cervical cancers in Scotland was no different from collated, worldwide estimates of around 70%. To detect a prevalence of HPV type 16/18 of 70% to within 10%, we needed a sample size of 323 using a two-sided 95% confidence interval (CI). To assess how representative our study population was, it was compared with the cancer registry data on 1752 cervical cancers diagnosed in Scotland from 2000–05 (http://www.scotland.gov.uk/Topics/Statistics/SIMD/BackgroundMethodology, 2009). Socioeconomic status and age at diagnosis were examined using *χ*^2^-tests.

Where data were available, the relationship between HPV 16 and/or 18 *vs* other or no HR-HPV and FIGO stages I–IV was assessed using a *χ*^2^-test. A similar analysis was performed to assess the relationship between HPV 16 and/or 18 positivity and ICD-O grade of differentiation (grades I–IV).

For mRNA expression analysis, frequency of type-specific transcripts was assessed and descriptive analysis was performed.

## Results

### Comparison of study population with national registry data

Of the 375 cervical cancers collated, 370 were included after data checking in the cancer registry. Omissions included two cases from women with permanent residence outside Scotland at time of diagnosis, two related to recurrent disease and one to a case of high-grade CIN. Using a *χ*^2^-test there was no evidence of a difference between socioeconomic status (*P*=0.273) or age (*P*=0.846) between the study population and the cancer registry data based on 1752 cases, indicating that our study population was representative. Of the 370 cases evaluated, 309 (83.5%) were squamous cell carcinoma, 54 (14.6%) were adenocarcinoma and 7 (1.9%) were ‘other’ types.

### HPV 16 and/or 18 prevalence

Comprehensive type-specific data on all DNA-positive cases are presented in Table 1. HPV DNA was detected in 325 out of 370 cases (88%) with HPV 16 in 207 cases, HPV 18 in 66 cases and HPV 16 and 18 in 8 cases. Overall, HPV 16 and/or 18 was detected in 72% (265 out of 370) of cases (67–76; 95% CI). Considering only HPV-positive cases, HPV 16 and/or 18 were detected in 82% (265 out of 325; 77–86 95% CI).

### Multiple infection and non-HPV 16/18 infection

Multiple infections were detected in 36 (10%) cases (95% CI 7–13%). Interestingly, by combining cases in Scotland's ‘central belt’ (Edinburgh, Glasgow and Monklands), we found evidence of a difference in the proportion of cases with multiple infections compared with non-central belt (Aberdeen and Dundee combined) with 7.8% more in the central belt with a 95% CI of (2–13.5), *P*=0.008. The three most prevalent types after 16 and 18 were 45, 33 and 31. A result of HPV X was generated quite frequently (23 appearances: 11 times in isolation, 12 as part of a multiple infection).

### Association between HPV 16/18 status and FIGO stage and grade

Table 2 depicts HPV 16/18 status *vs* non-HPV status according to FIGO stage, disease grade and age. There was no evidence from the available data of a relationship between HPV 16 and/or18 presence and either stage (*P*=0.561) or grade (*P*=0.553).

### HPV mRNA expression in cervical cancers

Of the 36 cervical cases in which multiple infections were detected, 32 proved adequate for expression analysis. In 25 out of 32 cases only one transcript was detected, in 6 out of 32 two transcripts were detected and in one case no transcript was detected. It was notable that in five cases (shaded), an RNA transcript was detected that was not reflected in the equivalent HPV DNA component. In the seven cases in which a low-risk type was detected (4 × HPV 6, 2 × HPV 68/73/97 and 1 × HPV 53), no transcripts were detected in five cases, and two cases (both HPV 6) were invalid for expression analysis. In the eleven cases in which HPV X was detected, one tested positive for HPV 18, no transcripts were detected in three cases, six cases were invalid for expression analysis and one case had insufficient material for re-testing. The two most commonly expressed transcripts were HPV 45 (number of appearances=12) and HPV 16 (number of appearances=11).

## Discussion

The HPV vaccine is being offered through a national programme in several parts of the world including Scotland. However, the vaccine is not a panacea for the eradication of HPV-associated cervical disease and is licensed only to protect against HPV types 16 and 18. Nevertheless, the findings of this study are very positive with 82% of a systematic collection of HPV DNA-positive ICC from across Scotland, being positive for HPV 16 and/or 18. A meta-analysis by Smith *et al* (2007) showed that HPV16/18 prevalence in Europe, North America and Australia was 74–77% of all cases tested. Thus, the Scottish data tally with this percentage. The next most frequently detected types in the Scottish ICC cases were, in order of prevalence, HPV 45, 33 and 31. Again this is an encouraging finding as there is increasing evidence to suggest a cross-protective effect with the bivalent vaccine for HPV types closely related to HPV 16 (HPV 31, 52 and possibly 33) and HPV 18 (HPV 45) (Jenkins, 2008).

It is notable that in our series 13 cancers (2.9%) occurred in women aged 18–24 years, with 11 out of 13 being infected with HPV 16 and/or 18. This is representative of collated Scottish Registry data from 2000 to 2005 in which 52 (2.96%) of 1752 cases were diagnosed in women <24 years. Routine cytology screening of women occurs in Scotland from age 20. Current discussion centres round the effectiveness of such a policy (Sasieni *et al*, 2009). Although we have not explored the screening history of the young women included in this study, early screening provides the potential for early detection and prevention of invasive disease.

It was interesting that in four cancers HPV 6 was the only HPV type detected. An explanation for this could be that there was a co-infecting high-risk type that was below the level of detection of the assay. However, in a recent review, which focussed on the classification of weakly carcinogenic HPV types, Schiffman *et al* (2009) suggested that ‘HPV 6, and other low-risk types can cause cancer in extremely rare virus–host circumstances’ .

We did not find an association between HPV 16 and/or 18 positivity and FIGO stage or disease grade. When the analysis was restricted to HPV 16 *vs* absence of HPV 16, again no association with stage of disease was found. Although this suggests that HPV type may have no substantive impact on prognosis, it is our intention to investigate this more directly by assessing survival according to HPV type in the study cohort as soon as sufficient follow-up time has accrued.

Preliminary data relating to transcript detection would indicate preferential expression of HPV 16 and interestingly HPV 45, the latter being expressed even when present in co-infections with HPV 16 and/or HPV 18. However, numbers are small and this phenomenon requires further investigation.

In conclusion, this evaluation would suggest that the bivalent prophylactic HPV vaccine will eventually have a significant impact on the reduction of ICC in Scotland. In addition, the accumulated data will act as a baseline against which future changes in type-specific prevalence in cancers can be referred as HPV immunisation embeds.

## Figures and Tables

**Table 1:**
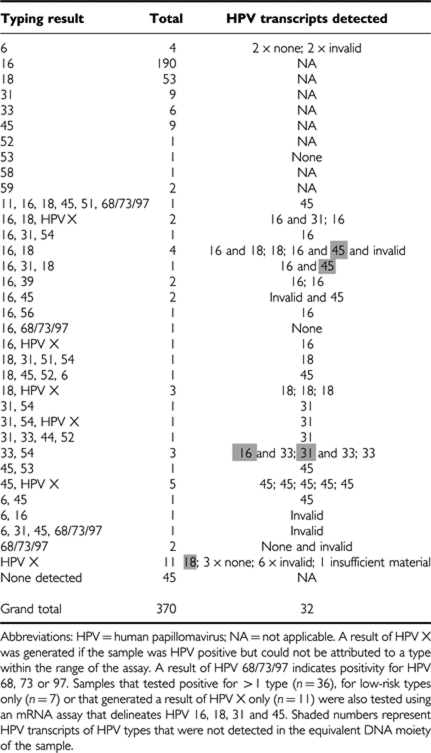
Overall distribution of HPV types in 370 invasive Scottish cancers

**Table 2:** HPV 16/18 status related to age, disease stage (FIGO) and disease grade

	**HPV16/18 *n* (%)**	**Not HPV16/18 *n* (%)**	**Total**
All	265 (71.6)	105 (28.4)	370
*FIGO*			
1	114 (73.1)	42 (26.9)	156
2	40 (78.4)	11 (21.6)	51
3	42 (73.7)	15 (26.3)	57
4	9 (60.0)	6 (40.0)	15
Not known	60 (65.9)	31 (34.1)	91
			
*Grade*			
1	8 (66.7)	4 (33.3)	12
2	71 (73.2)	26 (26.8)	97
3	75 (66.4)	38 (33.6)	113
4	1 (100.0)	0 (0.0)	1
Not known	110 (74.8)	37 (25.2)	147
			
*Age group (years)*			
<35	69 (80.2)	17 (19.8)	86
35–49	96 (76.2)	30 (23.8)	126
50–64	60 (74.1)	21 (25.9)	81
⩾65	40 (51.9)	37 (48.1)	77

Abbreviation: HPV=human papillomavirus.
